# Integrated MicroRNA-mRNA Analyses of the Osteogenic Differentiation of Human Dental Pulp Stem Cells by a Helioxanthin Derivative

**DOI:** 10.3390/cimb46100651

**Published:** 2024-09-28

**Authors:** Yasuyuki Fujii, Sakura Minami, Ayano Hatori, Yoko Kawase-Koga, Toru Ogasawara, Daichi Chikazu

**Affiliations:** 1 Department of Oral and Maxillofacial Surgery, Tokyo Medical University, 6-7-1 Nishishinjuku, Shinjuku-ku, Tokyo 160-0023, Japan; mnm81@tokyo-med.ac.jp (S.M.); hatori@uchc.edu (A.H.); koga.yoko@twmu.ac.jp (Y.K.-K.); chikazu@tokyo-med.ac.jp (D.C.); 2 Department of Oral and Maxillofacial Surgery, School of Medicine, Tokyo Women’s Medical University, 8-1 Kawadachou, Shinjuku-ku, Tokyo 160-0023, Japan; 3 Department of Oral and Maxillofacial Surgery, Graduate School of Medicine and Faculty of Medicine, University of Tokyo, 7-3-1 The Hongo, Bunkyo-ku, Tokyo 113-8655, Japan; togasawara-tky@umin.ac.jp

**Keywords:** osteogenic molecules, dental pulp stem cells, extracellular vesicles, microRNA

## Abstract

Dental pulp stem cells (DPSCs) demonstrate high proliferative and multilineage differentiation potential. As previously reported, the helioxanthin derivative 4-(4-methoxyphenyl)pyrido[40,30:4,5]thieno[2,3-b]pyridine-2-carboxamide (TH) has been demonstrated to induce the osteogenic differentiation of DPSCs. However, the mechanism of osteogenesis induced by TH in DPSCs remains unknown. The objective of this study was to identify functional extracellular vesicle (EV) microRNAs (miRNAs), and the principal genes involved in the TH-induced osteogenesis of DPSCs. DPSCs were derived from dental pulp extracted from the third molars of three healthy subjects, and were cultured with or without TH. miRNAs were extracted from DPSC-derived EVs. The gene expression patterns of mRNA and miRNA were compared using RNA-Seq and miRNA-Seq. To investigate miRNA/mRNA interacting networks, functional analyses were performed by Ingenuity Pathway Analysis. Alkaline phosphatase (ALP) staining demonstrated that treatment with TH resulted in enhanced ALP activity in DPSCs after 7 days. The expression levels of ALP and type 1 collagen alpha 1 were significantly higher in TH-induced DPSCs on day 7. RNA-Seq and miRNA-Seq analyses identified 869 differentially expressed genes (DEGs) and 18 miRNA-DEGs. Gene Ontology analysis of the mRNA-Seq results showed that TH induced several biological activities associated with signal transduction, cell adhesion, and cell differentiation. Integrated miRNA-mRNA analyses showed that these miRNAs contain the targeting information of 277 mRNAs of the DEGs. Among them, 17 target genes known to be involved in the differentiation of osteoblasts, and 24 target genes known to be involved in the differentiation of bone cells were identified. Quantitative real-time PCR showed that *WNT5a* expression in DPSCs was upregulated by 48 h of TH treatment. Upstream regulator analysis indicated that *WNT3a*, *FOS*, and *RAC1* may be responsible for gene expression changes in DPSCs after TH treatment. EV miRNA regulatory networks might play crucial roles in TH-induced osteogenic differentiation of DPSCs. Our results presented herein offer valuable insights that will facilitate further research into the mechanism of osteogenesis of DPSCs, which is expected to lead to the clinical application of TH-induced DPSCs for bone regeneration. Furthermore, EVs derived from TH-induced DPSCs might be useful as therapeutic tools for bone defects.

## 1. Introduction

Dental pulp stem cells (DPSCs) are easily isolated from extracted teeth and have been considered a promising cell source for regenerative medicine and tissue engineering. DPSCs demonstrate high proliferative and multilineage differentiation potential [1]. Many studies have documented the osteogenic differentiation capacity of DPSCs, as well as the underlying molecular mechanisms of osteogenic differentiation and bone regeneration involving DPSCs. As previously reported, the helioxanthin derivative 4-(4-methoxyphenyl)pyrido[40,30:4,5]thieno[2,3-b]pyridine-2-carboxamide (TH) has been demonstrated to induce the osteogenic differentiation of DPSCs [2,3,4]. TH has also been reported to induce the osteogenic differentiation of iPS cells and other cell lines, such as C3H10T1/2 cells and MC3T3-E1 cells [5,6,7]. On the other hand, TH suppresses osteoclast differentiation by stimulating nitric oxide production and inhibiting phosphodiesterase activity, both of which lead to the upregulation of intracellular cyclic guanosine monophosphate [8]. Thus, TH affects bone metabolism and has the potential as a novel antiosteoporotic agent for clinical application. Nevertheless, the precise mechanism by which TH affects bone metabolism remains unclear.

Extracellular vesicles (EVs) transport a variety of small biomolecules including microRNAs (miRNAs) to surrounding cells [9,10]. Previous studies have reported that EVs or miRNAs derived from EVs promote the osteogenic differentiation of mesenchymal stem cells (MSCs) and bone regeneration in vivo [11,12,13,14]. Thus, EVs and miRNAs derived from EVs play a vital role in the osteogenic differentiation of MSCs.

In this study, we hypothesized that miRNAs derived from EVs of TH-induced DPSCs promote the osteogenic differentiation of DPSCs. The objective of this study was to identify functional EV miRNAs and the principal genes involved in the TH-induced osteogenesis of DPSCs.

## 2. Materials and Methods

### 2.1. Isolation and Culture of DPSCs

This study was performed after receiving written informed consent from all subjects and was approved by the Institutional Ethics Committee of the Tokyo Medical University, Japan (study approval no.: T2020-0343; approved on 5 January 2021). DPSCs were derived from dental pulp extracted from the third molars of 3 healthy subjects at Tokyo Medical University Hospital. The cells were seeded at a density of 1 × 10⁵ onto 100 mm dishes, and cultured in control medium (CM; alpha minimum essential medium [αMEM, Gibco/BRL, Cheshire, UK] with 15% fetal bovine serum [FBS; Biowest, Nuaillé, France] and 1% penicillin–streptomycin–amphotericin B suspension [PSA; Wako Pure Chemical Industries, Osaka, Japan]), as previously described [2]. To induce osteogenic differentiation, DPSCs were seeded at a density of 1 × 104 onto 12-well plates, and cultured for 7 days in osteogenic differentiation medium (ODM;αMEM with 10% FBS, 1% PSA, 10 nM dexamethasone [Wako Pure Chemical Industries], 10 mM α-glycerophosphate [Sigma-Aldrich, Darmstadt, Germany], and 100 µM L-ascorbate-2-phosphate [Wako Pure Chemical Industries]). TH (Tokyo Chemical Industry, Tokyo, Japan) was dissolved in dimethyl sulfoxide (DMSO) and 10-6 M TH was added to CM or ODM.

### 2.2. Alkaline Phosphatase (ALP) Staining

DPSCs were cultured in ODM for 7 days and subsequently stained with ALP, as previously described [15]. The procedure involved a brief washing with phosphate-buffered saline, fixing in 70% ethanol, and staining with 0.01% naphthol AS-MX phosphate (Sigma-Aldrich) in the presence of 1% N,N-dimethyl formamide (Wako Pure Chemical Industries) as a substrate and 0.06% Fast Blue BB salt (Sigma-Aldrich) as a coupler for 10 min.

### 2.3. Extraction of DPSC-Derived EVs

EVs were isolated from the CM of DPSCs. Once 80% confluency was reached, the cells were cultured in FBS-free NM with DMSO (control) or TH for 2 days. The supernatants were collected and the DPSC-derived EVs were purified, as previously reported [16,17]. PureExo^®^ Exosome isolation kit (101Bio, Mountain View, CA, USA) was used for EV isolation, in accordance with the manufacturer’s instructions.

### 2.4. RNA Isolation and Quantitative Real-Time PCR (qPCR)

Total RNA from DPSCs was isolated using ISOGEN (Invitrogen, Carlsbad, MA, USA). Total RNA from DPSCs-EVs was isolated using QIAzol and miRNeasy Mini Kit (Qiagen, Hilden, Germany), in accordance with the manufacturer’s instructions. Reverse transcription was performed using the QuantiTect Reverse Transcription kit (Qiagen). qPCR was performed as described [2]. Data were normalized to GAPDH expression. Relative differences among samples were analyzed using the ΔΔCT method. The primer sequences used are presented in Table 1.

### 2.5. RNA Sequencing (RNA-Seq), miRNA Sequencing (miRNA-Seq), and Ingenuity Pathway Analysis (IPA)

To analyze the effects of TH on gene expression in DPSCs, total RNA and miRNA were collected from DPSCs cultured in CM with or without TH for 48 h and then sent to Rhelixa, Inc. (Tokyo, Japan) for sequencing.

Raw read counts were mapped to known mRNA or miRNA regions by featureCounts software (version 1.6.3) based on the human genome reference sequence (hg38). A |log_2_ fold change| of greater than 1, and a *p*-value of less than 0.05 calculated by the Benjamini and Hochberg method were used as the thresholds for evaluating the statistical significance of the gene expression differences. Differentially expressed genes (DEGs) were analyzed by Gene Ontology (GO) enrichment analysis using GOATOOLS software (version 1.1.6). Heat maps were created from the Z-scores of the normalized counts using stats (version 3.6.1) and gplots (version 3.0.1.1) R packages, and volcano plots were generated using plotly software (version 4.9.2.1). DEGs and miRNA-DEGs were analyzed by IPA (Qiagen).

### 2.6. Statistical Analysis

All statistical analyses were conducted using Prism 9 software (Version 9.3.0, GraphPad Software, San Diego, CA, USA). The experimental data are presented as the mean ± standard deviations (SD) of 3 samples. Differences between the control and TH groups have been evaluated by the paired Student *t*-test.

## 3. Results

### 3.1. Effects of TH on the Osteogenic Differentiation of DPSCs

**Figure 1:**
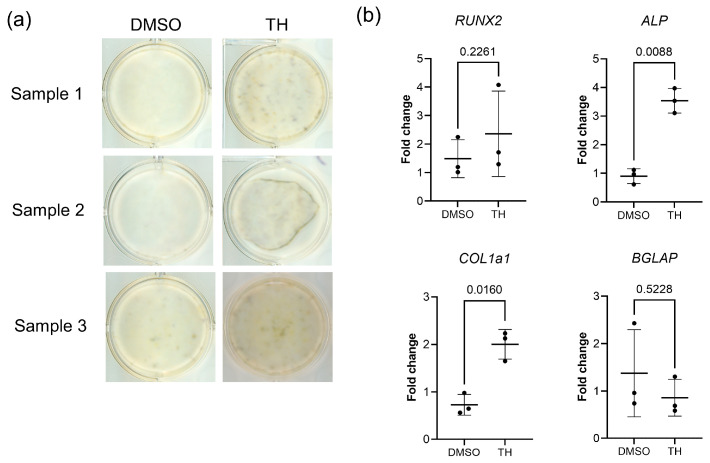
Effect of TH on the osteogenic differentiation ability of DPSCs. (**a**) ALP staining of DPSCs cultured in ODM with TH or with the DMSO control for 7 days (*n* = 3). (**b**) Expression level changes of osteogenic differentiation markers in DPSCs cultured in OM with TH or with the DMSO control for 7 days (*n* = 3). Error bars represent the SDs. Statistical analyses were performed using the paired *t*-test.

First, we investigated the effects of TH on the osteogenic differentiation of DPSCs. ALP staining demonstrated that TH augmented ALP activity in DPSCs after 7 days of treatment (Figure 1a). qPCR analyses demonstrated that TH-induced DPSCs demonstrated markedly increased expression levels of *ALP* (*p* = 0.088) and type 1 collagen alpha 1 (*COL1a1*; *p* = 0.160) on day 7 (Figure 1b). However, no statistically significant differences were observed in the expression levels of *Runx2* (*p* = 0.2261) and *BGLAP* (*p* = 0.5228). These findings imply that TH may facilitate the osteogenic differentiation of DPSCs.

### 3.2. mRNA and miRNA Expression Patterns in DPSCs Cultured with or without TH

**Figure 2:**
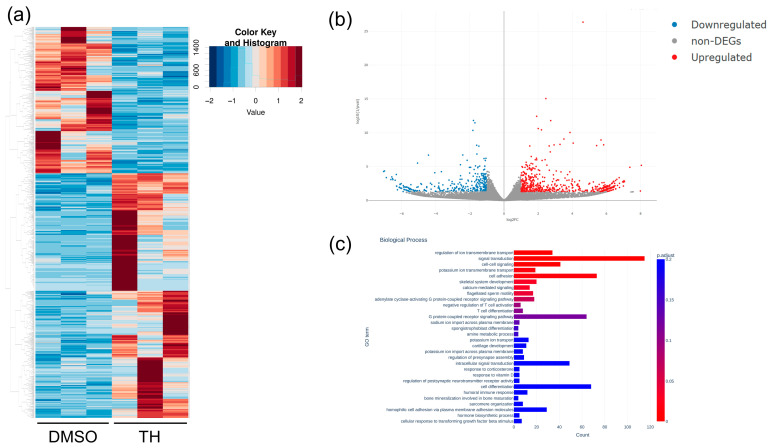
Comparison of gene expression profiles of DPSCs cultured with or without TH using RNA-Seq. (**a**) Heat map showing 869 DEGs between DPSCs cultured with and without TH. Red and blue indicate relative overexpressed and underexpressed genes, respectively. (**b**) Volcano plot showing DEG expression between DPSCs cultured with and without TH. Blue dots show the downregulated DEGs and red dots show the upregulated DEGs. The vertical axis of the graph indicates *p*-value and the horizontal axis indicates log2 fold change. (**c**) GO enrichment analysis. The 30 most significantly (*p* < 0.05) enriched GO terms of biological processes are presented.

**Figure 3:**
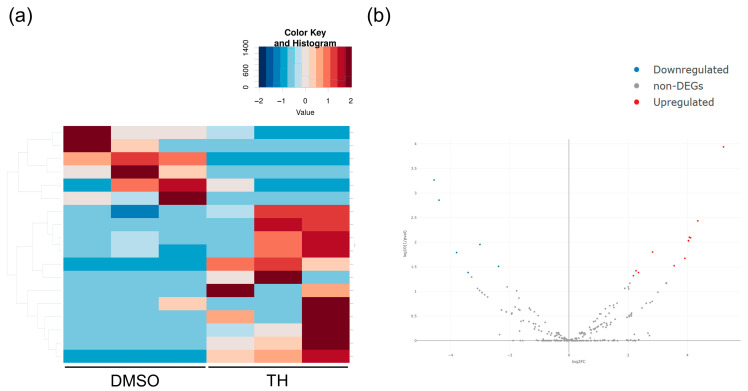
Comparison of gene expression profiles in DPSCs cultured with or without TH using miRNA-Seq. (**a**) Heat map showing 18 miRNA-DEGs between DPSCs cultured with and without TH. (**b**) Volcano plot showing miRNA-DEG expression between DPSCs cultured with and without TH. Blue dots show the downregulated DEGs and red dots show the upregulated DEGs. The vertical axis of the graph indicates *p*-value and the horizontal axis indicates log2 fold change. Blue dots show the downregulated miRNA-DEGs and red dots show the upregulated miRNA-DEGs.

Total RNA was isolated from DPSCs cultured in CM with or without TH, and mRNA expression was analyzed using RNA-Seq. RNA-Seq showed 869 DEGs (Figure 2a,b). Furthermore, GO analysis of the mRNA-Seq results showed that TH induced some biological activities associated with signal transduction, cell adhesion, and cell differentiation (Figure 2c). In addition, miRNAs were isolated from EVs derived from DPSCs cultured in CM with or without TH, and miRNA expression was analyzed using miRNA-Seq. The results showed 18 miRNA-DEGs (Figure 3a,b).

### 3.3. Predicted mRNA and Pathways Targeted by EV-Derived miRNAs of TH-Induced DPSCs

**Figure 4:**
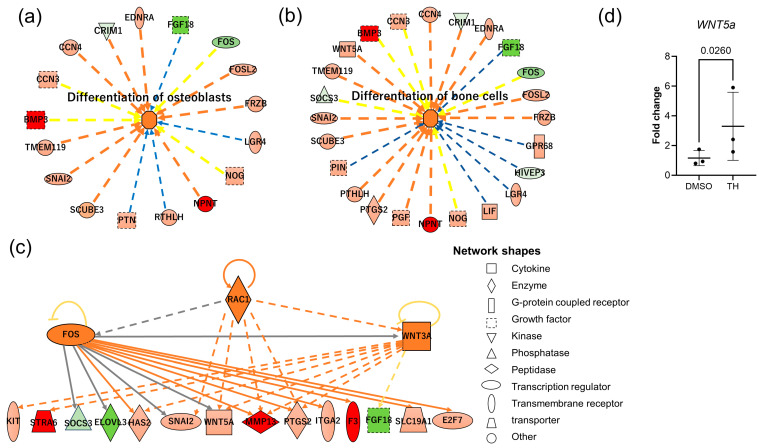
IPA analysis of mRNA-Seq and miRNA-Seq data. (**a**,**b**) Disease and biological function analyses of the DEGs for 2 biological functions (differentiation of osteoblasts and differentiation of bone cells). (**c**) Upstream regulator analysis. Upstream regulators and their targets displayed as a network of interactions with their respective expression trends. In the upper tier, there are 3 upstream regulators (RAC1, WNT3a, and FOS) predicted to be activated (orange color). In (**a**–**c**), upregulated and downregulated genes are highlighted in red and green, respectively, and the color depth is correlated to the fold change. The lines indicate the predicted relationship between nodes and biological function: orange representing activation, yellow representing an inconsistent (activation or inhibition) effect, and gray representing an effect that was not predicted. Solid and dashed lines between genes represent known direct and indirect gene interactions, respectively. The octagonal symbol defines function, with orange representing activation. (**d**) Expression levels of *WNT5a* cultured in CM with TH or with DMSO for 48 h (*n* = 3). Error bars represent the SDs. Statistical analyses were performed using the ratio paired *t*-test.

To investigate the specific miRNA/mRNA-interacting network, mRNA-Seq and miRNA-Seq data were analyzed, and functional analyses were performed using IPA. Integrated miRNA-mRNA analyses showed that these miRNAs have targeting information for 277 mRNAs of the DEGs. Among them, 17 target genes known to be involved in the differentiation of osteoblasts, and 24 target genes known to be involved in the differentiation of bone cells were identified (Figure 4a,b). Upstream regulator analysis indicated that RAC1, WNT3a, and FOS may be responsible for gene expression changes in DPSCs after TH stimulation (Figure 4c). qPCR data showed that *WNT5a* was significantly upregulated in DPSCs at 48 h of TH treatment (Figure 4d; *p* = 0.0260). These results suggest that the induction of osteogenesis by TH in DPSCs is regulated by EV-derived miRNAs, and might also involve Wnt5a signaling.

## 4. Discussion

Both canonical Wnt–β-catenin pathway and noncanonical Wnt pathways regulate bone turnover, mainly by promoting osteoblast differentiation and indirectly controlling osteoclastogenesis [18,19]. Wnt5a-induced noncanonical Wnt signaling promotes the differentiation of mesenchymal stem cells into osteoblast lineage cells [20]. It was also previously reported that recombinant WNT5A enhances osteo/odontogenic differentiation in DPSCs [21]. Previous studies have also reported the importance of WNT5a during tooth development. Wnt5a-deficient mice exhibit retarded tooth development [22]. Wnt5a promotes differentiation of human dental papilla cells [23], but attenuates Wnt3a-induced alkaline phosphatase expression in dental follicle cells [24]. These results suggest that WNT5a signaling plays a crucial role in the proliferation and differentiation of the cells in dental pulp, while previous studies have reported that some miRNAs have been reported to target WNT5A [25,26]. In this study, miRNA-Seq showed 18 miRNA-DEGs, and these 18 miRNAs upregulated by TH might directly or indirectly regulate the osteogenic differentiation of DPSCs through Wnt signaling. Upstream regulator analysis in our study further suggests that RAC1, WNT3a, and FOS may be responsible for gene expression changes in DPSCs after TH stimulation. RAC1 transmits signals originating from the Wnt signaling pathway [27], and the absence of Rac1 in preosteoblasts diminishes their osteoblastic differentiation [28]. Also, a previous study reported that WNT3a induced the gene expressions in early stages of osteoblastogenesis while inhibiting genes expressed in later stages [29]. The importance of the Fos family in bone metabolism has been reported, and the changes of these expression lead to bone diseases such as osteopetrosis, osteosarcoma, and osteosclerosis [30,31]. Future studies will investigate how these gene expressions affects osteogenesis or EV functions in DPSCs.

Not only TH, but other xanthine derivatives also affect bone metabolism. For example, the xanthine derivative 7-[2-[4-(2-chlorophenyl)piperazinyl]ethyl]-1,3-dimethylxanthine (KMUP-1) promotes osteoblast differentiation through the adenosine 3′,5′-cyclic monophosphate and guanosine 3′,5′-cyclic monophosphate pathways, and signaling of BMP-2/Smad1/5/8 and Wnt/β-catenin [32]. However, little is known about the mechanisms underlying the effects of these xanthine derivatives on bone metabolism. Our results of miRNA-RNA analyses indicated that EV miRNAs might play an important role in the TH-induced osteogenesis of DPSCs. Toward the clinical application of TH-induced DPSCs for the regeneration of bone tissue, potential targets of the 18 miRNA-DEGs identified in our miRNA-Seq and the signaling pathways involved in regulating osteogenic differentiation should be investigated in the future. Furthermore, EVs are increasingly being expected as a therapeutic agent of tissue repair and regeneration [33]. EVs derived from TH-induced DPSCs might be tools in therapy for bone defects.

## 5. Conclusions

In conclusion, the findings of our present study indicate that TH stimulates the osteogenic differentiation of DPSCs, by modifying the expressions of EV miRNA. Integrated microRNA-mRNA analyses indicated that regulatory networks of EV-derived miRNA may play an important role in the osteogenic differentiation of DPSCs induced by TH. Furthermore, EV-derived miRNAs upregulated by TH might directly or indirectly regulate the osteogenic differentiation of DPSCs through Wnt signaling. Our results presented herein offer valuable insights that will facilitate further research into the mechanism of osteogenesis of DPSCs, which may enable the clinical application of TH-induced DPSCs or EV therapy derived from DPSCs for the regeneration of bone tissue in the future.

## Figures and Tables

**Table 1:** Primer sequences used for qPCR.t.

Gene	Primer Sequences (Forward [top] and Reverse [bottom], 5′-3′)	Accession No.
*ALP*	ATGAAGGAAAAGCCAAGCAG ATGGAGACATTCTCTCGTTC	NM_000478
*BGLAP*	GGCAGCGAGGTAGTGAAGAG AGCAGAGCGACACCCTAGAC	NM_199173
*COLIA1*	GTGCTAAAGGTGCCAATGGT CTCCTCGCTTTCCTTCCTCT	NM_000088
*GAPDH*	GAAGGTGAAGGTCGGAGTCA GAAGATGGTGATGGGATTTC	BC023632

## Data Availability

The data presented in this study are available from the corresponding author upon reasonable request.
